# Documented Rheumatic Disease and Post-Discharge Mortality After Acute Coronary Syndrome: A Two-Center Registry Study

**DOI:** 10.3390/medicina62071306

**Published:** 2026-07-06

**Authors:** Ivana Jurin, Stela Hrkač, Goran Šukara, Irzal Hadžibegović, Karlo Gjuras, Andrija Matijević, Diana Rudan, Šime Manola, Denis Došen, Kristina Marić Bešić, Joško Mitrović

**Affiliations:** 1 Department of Cardiovascular Diseases, University Hospital Dubrava, 10000 Zagreb, Croatiadrudan3@yahoo.com (D.R.); sime.manola@icloud.com (Š.M.); 2 Division of Clinical Immunology, Allergology and Rheumatology, Department of Internal Medicine, University Hospital Dubrava, 10000 Zagreb, Croatia; 3 Faculty of Dental Medicine and Health Care, Josip Juraj Strossmayer University of Osijek, 31000 Osijek, Croatia; 4 Department of Family Medicine, Health Centre Bjelovar-Bilogora County, 43000 Bjelovar, Croatia; karlogjuras4@gmail.com; 5 Department of Cardiology, Sisters of Charity University Hospital Centre, 10000 Zagreb, Croatia; andmatijevic97@gmail.com; 6 University Center Varaždin, University North, 42000 Varaždin, Croatia; 7 School of Medicine, University of Zagreb, 10000 Zagreb, Croatia; kmaricbesic@gmail.com; 8 Department of Cardiovascular Diseases, University Hospital Centre Zagreb, 10000 Zagreb, Croatia; denisdosen@gmail.com; 9 Faculty of Pharmacy and Biochemistry, University of Zagreb, 10000 Zagreb, Croatia

**Keywords:** acute coronary syndrome, rheumatic diseases, psychiatric comorbidity, mortality, registries

## Abstract

*Background and Objectives*: Rheumatic diseases confer excess cardiovascular risk, yet prognosis after acute coronary syndrome (ACS) in contemporary angiography-treated care remains incompletely characterized, particularly when psychiatric multimorbidity is considered. We evaluated whether documented rheumatic disease was associated with psychiatric comorbidity and post-discharge mortality after ACS. *Materials and Methods*: We retrospectively analyzed a predefined two-center registry extract of 2950 consecutive patients who underwent coronary angiography for ACS. Documented rheumatic disease was identified from diagnoses recorded in admission history, prior medical records, or discharge documentation and was not re-adjudicated. The primary outcome was post-discharge all-cause mortality. *Results*: Documented rheumatic disease was present in 106 patients (3.6%). Compared with patients without documented rheumatic disease, exposed patients were older, more often women, more often hypertensive, and more likely to have a documented psychiatric disorder (25.5% vs. 14.1%). Short-term mortality was similar, whereas crude overall long-term mortality was higher (27.4% vs. 19.3%). Among hospital survivors with usable follow-up, post-discharge survival was worse (log-rank *p* = 0.013). Documented rheumatic disease was associated with higher post-discharge mortality in unadjusted analysis (hazard ratio 1.66, 95% confidence interval 1.11–2.48) and in a prespecified parsimonious model (adjusted hazard ratio 1.56, 95% confidence interval 1.04–2.34); the association attenuated and was no longer statistically significant in a broader exploratory model (adjusted hazard ratio 1.35, 95% confidence interval 0.87–2.07). Documented psychiatric disorder independently predicted mortality. *Conclusions*: In angiography-treated ACS, documented rheumatic disease was associated with greater psychiatric comorbidity and worse post-discharge survival in a small, documentation-defined, heterogeneous subgroup. Because the signal attenuated in broader exploratory adjustment and exposure ascertainment was documentation-based, the findings should be regarded as hypothesis-generating rather than disease-specific or causal.

## 1. Introduction

Patients with chronic rheumatic diseases carry excess cardiovascular risk because conventional risk factors interact with persistent inflammation, immune activation, endothelial dysfunction, prothrombotic pathways, and treatment-related effects [1,2,3]. This excess risk is best documented in rheumatoid arthritis and systemic lupus erythematosus, but it also extends to gout, spondyloarthritis, systemic sclerosis, vasculitis, and related inflammatory disorders [4,5,6,7,8,9,10,11,12]. What remains less well defined is how these patients fare once they enter contemporary ACS pathways built around early angiography, revascularization, and guideline-directed secondary prevention [13].

That unanswered question matters clinically because the vulnerable phase may no longer be the admission itself. Recent cohort studies and meta-analytic data suggest that, in inflammatory rheumatic disease, excess cardiovascular risk is often expressed more clearly during longitudinal follow-up than during the index coronary hospitalization [14,15,16,17,18,19,20]. Modern ACS care may narrow early differences, while the period after discharge remains exposed to multimorbidity, treatment complexity, and fragmented follow-up.

Psychiatric comorbidity may be one reason why recovery diverges. Depression and anxiety are more common in rheumatoid arthritis and other immune-mediated inflammatory diseases than in the general population [21,22], and post-myocardial infarction psychological distress is common, prognostically relevant, and still incompletely addressed in cardiovascular guidance [23,24,25,26,27]. In patients with documented rheumatic disease, psychiatric burden could worsen post-ACS recovery through poorer adherence, lower participation in rehabilitation and lifestyle change, delayed help-seeking, social vulnerability, sleep disturbance, and interacting inflammatory or neurohumoral pathways. Yet real-world studies that examine psychiatric burden alongside post-discharge survival within contemporary ACS pathways remain limited.

We therefore analyzed a two-center real-world registry of consecutive patients undergoing coronary angiography for ACS. Our primary objective was to evaluate whether documented rheumatic disease was associated with post-discharge all-cause mortality after ACS. Secondary objectives were to describe the burden of documented psychiatric disorder in this subgroup and to explore whether the observed signal appeared to reflect a documentation-defined multimorbid rheumatic subgroup in practice rather than a disease-specific effect attributable to any single rheumatic diagnosis.

## 2. Materials and Methods

We performed a retrospective cohort analysis of a predefined extract from an observational ACS registry maintained at two Croatian tertiary centers. The registry captures consecutive hospitalizations for ST elevation myocardial infarction and non-ST elevation ACS treated with coronary angiography; in registry coding, the non-ST elevation category comprised non-ST elevation myocardial infarction and unstable angina. In the current extract, index admissions spanned 18 December 2016 to February 2025, and follow-up was censored at the last available registry contact recorded in the dataset. Reporting follows the Strengthening the Reporting of Observational Studies in Epidemiology (STROBE) statement [15].

The exposure of interest was documented rheumatic disease in the medical record, identified from diagnoses recorded in admission history, past medical history, discharge summaries, and other physician-entered clinical documentation within the registry extract rather than from re-adjudicated case review or a uniform International Classification of Diseases (ICD)-only algorithm. Documented rheumatic disease was treated as a binary variable and included gout, rheumatoid arthritis, spondyloarthritis, systemic lupus erythematosus, systemic sclerosis, vasculitis, and related diagnoses. Because ascertainment was registry-based, latent or later-diagnosed rheumatic disease could not be captured, and baseline diagnoses were not re-adjudicated by a rheumatology review panel. Accordingly, the analysis was designed to evaluate the prognosis of a documentation-defined heterogeneous rheumatic subgroup encountered in practice, not to provide a disease-specific causal estimate for all rheumatic diseases or specifically for inflammatory rheumatic disease alone. Documented psychiatric disorder was defined pragmatically as any psychiatric diagnosis recorded in past medical history or discharge documentation, including mood, anxiety, psychotic, bipolar-spectrum, psycho-organic, and other psychiatric diagnoses when explicitly recorded. The registry did not store a uniform psychiatric taxonomy, and we could not reliably distinguish previously established diagnoses from diagnoses newly documented during the index admission. Thyroid-stimulating hormone values were analyzed only as a secondary exploratory variable when available from routine admission laboratory testing.

The primary time-to-event outcome was post-discharge all-cause mortality. Patients who died during the index hospitalization were retained for descriptive short-term analyses but were excluded from post-discharge survival analyses. Hospital survivors were followed from discharge until death or last recorded contact; deaths were identified from registry follow-up/status fields and corresponding medical-record follow-up entries captured in the registry extract. Because cause-of-death adjudication was not available in a sufficiently complete and standardized form, mortality analyses were limited to all-cause death. Survival analyses were restricted to hospital survivors with non-missing, non-negative follow-up duration; the unadjusted post-discharge dataset comprised 2818 patients (102 with documented rheumatic disease and 2716 without), corresponding to 99.8% completeness among hospital survivors. Reverse Kaplan–Meier median follow-up was 1822 days; observed follow-up time among included hospital survivors had a median of 1260 days. Secondary descriptive outcomes were in-hospital, 30-day, and 1-year all-cause mortality. Prescription of aspirin, statins, and beta-blockers at discharge was also described. A broader composite cardiovascular endpoint was analyzed descriptively but was not the primary focus of the present report.

Continuous variables are reported as median with interquartile range and categorical variables as the number with percentage. Because key continuous variables showed skewed or bounded distributions on inspection, we used nonparametric summaries and between-group comparisons throughout rather than assuming normality. Between-group comparisons used the Mann–Whitney U test, chi-square test, or Fisher’s exact test, as appropriate. Post-discharge survival was examined with Kaplan–Meier estimates with pointwise 95% confidence bands and log-rank testing, and Cox proportional hazards models are reported as hazard ratios with 95% confidence intervals. The primary multivariable model was intentionally parsimonious and adjusted a priori for age, sex, arterial hypertension, diabetes mellitus, smoking history, ACS type, and left ventricular ejection fraction. An expanded exploratory model additionally adjusted for admission creatinine and prior myocardial infarction or prior percutaneous coronary intervention. eGFR was not entered together with creatinine because of collinearity and greater missingness. A prespecified sensitivity analysis repeated survival models after exclusion of gout-containing diagnoses because gout accounted for approximately one third of documented rheumatic disease cases and may differ clinically from systemic autoimmune/inflammatory disorders. Logistic regression was used to examine the association between documented rheumatic disease and documented psychiatric disorder. The proportional hazards assumption was checked by visual inspection of Kaplan–Meier separation and complementary log-log plots and showed no major apparent violation. Analyses used available-case denominators; model-specific N/events are reported with each model in the Results. 

### Ethics

The study was conducted in accordance with the Declaration of Helsinki and approved by the institutional ethics committees of both participating centers. The requirement for individual informed consent was waived because this was a retrospective analysis of routinely collected registry data.

## 3. Results

Documented rheumatic disease was present in 106 of 2950 patients (3.6%). The most frequent diagnoses were gout (34.0%), rheumatoid arthritis (28.3%), other spondyloarthritis (11.3%), and systemic lupus erythematosus (6.6%). Compared with patients without documented rheumatic disease, exposed patients were older (68 [61–74] vs. 64 [56–73] years; *p* = 0.017), more often women (46.2% vs. 30.2%; *p* < 0.001), more often suffering from arterial hypertension (86.8% vs. 73.9%; *p* = 0.003), and more often had a documented psychiatric disorder (25.5% vs. 14.1%; *p* = 0.001). Baseline characteristics are summarized in Table 1, and the spectrum of documented rheumatic diagnoses is shown in Table 2. Given the heterogeneity of the exposure and the prominence of gout, the main comparison should be interpreted as applying to this documentation-defined rheumatic subgroup rather than to a single inflammatory rheumatic disease entity.

Continuous variables are presented as median [interquartile range]. Variables with incomplete capture included body mass index (2823/2950), hemoglobin (2853/2950), red cell distribution width (2851/2950), left ventricular ejection fraction (2756/2950), and creatinine (2856/2950). “Optimal medical treatment only” denotes conservative management after diagnostic angiography without PCI or CABG during the index episode.

Selected admission laboratory findings suggested a more multimorbid clinical profile in the documented rheumatic disease group, including lower hemoglobin (137 [125–146] vs. 144 [133–154] g/L; *p* < 0.001) and higher red cell distribution width (13.9 [13.3–15.0] vs. 13.5 [13.0–14.1]%; *p* < 0.001), whereas admission creatinine was not higher and median left ventricular ejection fraction was similar. Admission thyroid-stimulating hormone values, available in 1838 of 2950 patients (62.3%), were nearly identical in both groups (1.9 [1.1–3.2] vs. 1.9 [1.1–3.2]; *p* = 0.514). Among hospital survivors with available discharge medication fields, prescription rates of aspirin, statins, and beta-blockers were numerically similar between groups.

Short-term mortality did not differ significantly between groups: in-hospital mortality was 3.8% in patients with documented rheumatic disease and 4.3% in patients without documented rheumatic disease, 30-day mortality was 4.7% and 5.6%, and 1-year mortality was 9.4% and 9.0%, respectively. In the full cohort, crude overall long-term mortality was higher in patients with documented rheumatic disease (29/106 [27.4%] vs. 550/2844 [19.3%]; *p* = 0.039). Mortality outcomes are summarized in Table 3.

**Figure 1:**
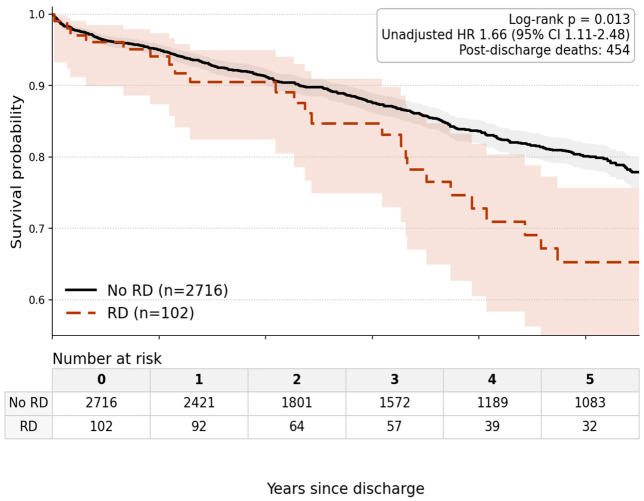
Kaplan–Meier estimates of post-discharge all-cause mortality in patients with documented rheumatic disease and patients without documented rheumatic disease. Shaded bands denote pointwise 95% confidence intervals. Log-rank *p* = 0.013.

Among hospital survivors with usable follow-up (102 with documented rheumatic disease and 2716 without), post-discharge survival was worse in patients with documented rheumatic disease (log-rank *p* = 0.013; Figure 1). In unadjusted Cox analysis, documented rheumatic disease was associated with higher post-discharge mortality (hazard ratio 1.66, 95% confidence interval 1.11–2.48). The association remained significant in the prespecified parsimonious model (adjusted hazard ratio 1.56, 95% confidence interval 1.04–2.34), but attenuated and was no longer statistically significant in the expanded exploratory model (adjusted hazard ratio 1.35, 95% confidence interval 0.87–2.07). In sensitivity analysis after exclusion of gout-containing diagnoses, the signal remained present in the parsimonious model (adjusted hazard ratio 1.78, 95% confidence interval 1.11–2.87) and attenuated in the expanded model (adjusted hazard ratio 1.38, 95% confidence interval 0.82–2.33). Accordingly, the adjusted findings support a modest, model-sensitive association rather than a robust disease-specific independent effect.

**Figure 2:**
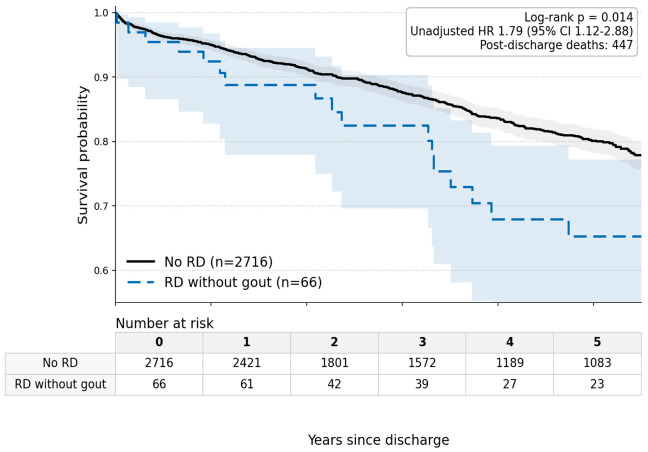
Kaplan–Meier estimates of post-discharge all-cause mortality after exclusion of gout-containing diagnoses. Shaded bands denote pointwise 95% confidence intervals. Log-rank *p* = 0.014.

In adjusted logistic regression, documented rheumatic disease remained associated with documented psychiatric disorder (adjusted odds ratio 1.96, 95% confidence interval 1.24–3.08; *p* = 0.004). In a Cox model that included both documented rheumatic disease and documented psychiatric disorder, documented psychiatric disorder independently predicted post-discharge mortality (adjusted hazard ratio 1.85, 95% confidence interval 1.49–2.31). These analyses were intended to describe co-occurring psychiatric burden and its prognostic relevance; they do not establish mediation, and formal interaction testing between documented rheumatic disease and documented psychiatric disorder was not significant. Survival plots and regression analyses are presented in Figure 1 and Figure 2, and Table 4.

**Table 4:** Regression analyses.

Analysis	Model	N/Events	Estimate (95% CI)	*p* Value
Documented rheumatic disease vs. none	Unadjusted Cox	2818/454	HR 1.66 (1.11–2.48)	0.014
Documented rheumatic disease vs. none	Parsimonious adjusted Cox *	2678/443	HR 1.56 (1.04–2.34)	0.031
Documented rheumatic disease vs. none	Expanded adjusted Cox ^†^	2614/431	HR 1.35 (0.87–2.07)	0.178
Non-gout documented rheumatic disease vs. none	Parsimonious adjusted Cox *	2646/436	HR 1.78 (1.11–2.87)	0.018
Non-gout documented rheumatic disease vs. none	Expanded adjusted Cox ^†^	2584/424	HR 1.38 (0.82–2.33)	0.226
Documented rheumatic disease → documented psychiatric disorder	Adjusted logistic regression ^‡^	2949/429	OR 1.96 (1.24–3.08)	0.004
Documented psychiatric disorder → mortality	Adjusted Cox with documented rheumatic disease ^§^	2678/443	HR 1.85 (1.49–2.31)	<0.001

* Adjusted for age, sex, arterial hypertension, diabetes mellitus, smoking history, acute coronary syndrome type, and left ventricular ejection fraction. ^†^ Expanded exploratory model additionally adjusted for admission creatinine and prior myocardial infarction or prior percutaneous coronary intervention. ^‡^ Adjusted for age and sex. ^§^ Adjusted for documented rheumatic disease, age, sex, arterial hypertension, diabetes mellitus, smoking history, acute coronary syndrome type, and left ventricular ejection fraction.

## 4. Discussion

Our central finding is clinically simple: in a contemporary angiography-treated ACS cohort, documented rheumatic disease did not primarily mark a more hazardous hospitalization, but a more fragile recovery after discharge. Compared with patients without documented rheumatic disease, these patients had a substantially higher burden of documented psychiatric disorder and worse post-discharge survival. The mortality signal persisted in the prespecified parsimonious model and in the non-gout sensitivity analysis, but attenuated and lost conventional statistical significance in the broader exploratory model. Therefore, attenuation is important. It argues against a strong independent disease effect that is robust to broader covariate adjustment and supports a more cautious interpretation: documented rheumatic disease functions here mainly as a marker of a clinically vulnerable, multimorbid recovery phenotype, not as proof of a causal mechanism.

The timing of the signal matters. Prior work established that inflammatory rheumatic disease is associated with excess cardiovascular risk overall, whereas more recent cohorts suggest that the divergence may become more apparent after the index coronary admission than during it [4,5,6,7,8,9,10,16,17,21,22,23,24,25]. Our data fit that pattern. In-hospital and early mortality were not materially different, but the separated survival curves implied that prognosis may be shaped less by the technical success of acute coronary treatment alone and more by what happens during rehabilitation, medication persistence, inflammatory disease control, and continuity of follow-up.

Several mechanisms may plausibly contribute to this delayed divergence. Residual inflammation, fluctuating disease activity, glucocorticoid exposure, renal and metabolic comorbidity, polypharmacy, frailty, and competing symptom burdens are unlikely to exert their full effect within the first days of hospitalization, but may accumulate over months and years. The heterogeneity of our documentation-defined rheumatic disease group is therefore both a limitation and a clinically recognizable reality: different diagnoses may operate through distinct pathways yet converge on a shared phenotype of multimorbid recovery after ACS. Importantly, the present data should not be interpreted as providing the same prognostic estimate for all rheumatic diseases, nor as proving that the signal is confined to inflammatory rheumatic disease only. Rather, the non-gout sensitivity analysis indicates that gout alone does not fully account for the association, while the small exposed sample precludes reliable disease-specific inference.

The psychiatric findings sharpen the clinical meaning of the study. Psychiatric disorders were substantially more common in the rheumatic disease subgroup and, across the whole cohort, documented psychiatric disorder independently predicted post-discharge mortality. Although mediation cannot be inferred from these analyses, the association is clinically plausible. Depression, anxiety, and broader post-myocardial infarction psychological distress are linked to poorer medication adherence, lower participation in cardiac rehabilitation, less durable lifestyle change, social vulnerability, and worse long-term cardiovascular prognosis [13,14,28,29,30]. Seen through that lens, psychiatric burden is not a side note to the coronary event; it is part of the recovery context that may help explain why some patients survive the admission but recover less well thereafter.

The clinical implication is straightforward: post-ACS care in patients with documented rheumatic disease should be organized as multimorbidity management rather than coronary follow-up alone. For cardiologists, a rheumatic disease label should trigger a broader recovery assessment, not reassurance based only on technically successful acute treatment. For rheumatologists, an ACS admission should prompt renewed attention to inflammatory control, medication burden, glucocorticoid exposure, and gout management where relevant. A pragmatic cardio-rheumatology pathway should also include optimization of secondary prevention and a low threshold for psychological or psychiatric assessment when adherence, recovery, or rehabilitation engagement appears fragile [26,27,29,30]. The aim is not to multiply appointments, but to align specialties around the phase of care in which prognosis begins to diverge.

The take-home message is clinical rather than mechanistic. This study does not prove that rheumatic disease itself causes worse long-term outcomes after ACS. It does, however, identify a recognizable documentation-defined subgroup whose recovery appears more complex, more psychiatrically burdened, and less resilient after discharge. That is a usable message for practice: technically successful acute coronary care should not close the risk conversation in patients with documented rheumatic disease. Future prospective studies should test whether integrated cardio-rheumatology follow-up, ideally coupled with structured attention to psychiatric vulnerability, medication burden, and post-discharge rehabilitation, can narrow this survival gap.

### Limitations

Several limitations should temper interpretation. Both documented rheumatic disease and documented psychiatric disorder were ascertainment-by-documentation variables and may have been under-recorded or misclassified. This approach may also miss patients with latent or initially undocumented rheumatic disease who receive a diagnosis later during follow-up. The exposed subgroup was small (106 patients overall and 102 entering post-discharge survival analyses), clinically heterogeneous, and underpowered for disease-specific subgroup inference, with gout accounting for one third of rheumatic disease cases. Psychiatric comorbidity was defined broadly from documentation rather than from a uniform taxonomy, and we could not reliably separate previously established psychiatric diagnoses from diagnoses newly documented during the index admission. Although hemoglobin, CRP, angiographic management, and selected discharge medication fields were available descriptively, stable multivariable adjustment remained constrained by the size of the exposed subgroup, and several other clinically relevant post-ACS prognostic factors were unavailable or not harmonized sufficiently for modeling, including Killip/severity measures, frailty, cardiac rehabilitation participation, immunosuppressive therapy, glucocorticoid burden, and standardized rheumatic disease activity metrics. Because eGFR was more incomplete and collinear with creatinine, it was not modeled together with creatinine. We also lacked sufficiently complete harmonized cause-specific mortality data; accordingly, mortality analyses were limited to all-cause death. The registry analysis was retrospective, follow-up completeness depended on the available registry timing fields, and inclusion was restricted to angiography-treated ACS patients, which may limit generalizability to more conservatively managed ACS populations. Finally, attenuation in the broader exploratory model indicates that residual confounding remains a real possibility and that the present analyses are better viewed as hypothesis-generating than definitive.

## 5. Conclusions

In a contemporary angiography-treated ACS registry, documented rheumatic disease was associated with greater documented psychiatric comorbidity, higher crude overall mortality, and worse post-discharge survival in a small, documentation-defined, heterogeneous subgroup. The association with post-discharge mortality persisted in a prespecified parsimonious model but attenuated and lost statistical significance in broader exploratory adjustment. The main practical message is therefore not that rheumatic disease itself causes worse long-term survival, but that after ACS, these patients appear to recover from a multimorbid episode in which inflammatory disease, psychiatric burden, and secondary prevention intersect. Because exposure ascertainment was documentation-based, the exposed subgroup was small and heterogeneous, and residual confounding cannot be excluded, the findings should be regarded as hypothesis-generating.

## Figures and Tables

**Table 1:** Selected baseline characteristics and admission laboratory findings.

Variable	Documented Rheumatic Disease (*n* = 106)	No Documented Rheumatic Disease (*n* = 2844)	*p* Value
Age, years	68 [61–74]	64 [56–73]	0.017
Female sex	49 (46.2%)	858 (30.2%)	<0.001
Arterial hypertension	92 (86.8%)	2101 (73.9%)	0.003
Diabetes mellitus	28 (26.4%)	704 (24.8%)	0.698
Smoking history	47 (44.3%)	1487 (52.3%)	0.108
Prior myocardial infarction	18 (17.0%)	358 (12.6%)	0.183
Prior percutaneous coronary intervention	19 (17.9%)	341 (12.0%)	0.067
Documented psychiatric disorder	27 (25.5%)	402 (14.1%)	0.001
Left ventricular ejection fraction, %	51 [45–60]	52 [45–60]	0.571
Creatinine, µmol/L	77 [65–100]	84 [71–101]	0.020
ST elevation myocardial infarction	58 (54.7%)	1560 (54.9%)	0.629
Optimal medical treatment only	13 (12.3%)	200 (7.0%)	0.041
Dyslipidemia	74 (69.8%)	2044 (71.9%)	0.724
Prior stroke/TIA	6 (5.7%)	179 (6.3%)	0.952
Peripheral artery disease	18 (17.0%)	372 (13.1%)	0.309
Body mass index, kg/m^2^	28.9 [25.7–32.5]	28.4 [25.7–31.4]	0.520
Hemoglobin, g/L	137 [125–146]	144 [133–154]	<0.001
Red cell distribution width, %	13.9 [13.3–15.0]	13.5 [13.0–14.1]	<0.001

**Table 2:** Spectrum of documented rheumatic diagnoses.

Diagnosis	*n*	% of Exposed Cohort
Gout	36	34.0
Rheumatoid arthritis	30	28.3
Other spondyloarthritis	12	11.3
Systemic lupus erythematosus	7	6.6
Psoriatic arthritis	5	4.7
Systemic sclerosis	3	2.8
Undifferentiated connective tissue disease/collagenosis	3	2.8
Polymyalgia rheumatica	2	1.9
Other vasculitides	2	1.9
Other single diagnoses	6	5.7

**Table 3:** Mortality outcomes.

Outcome	Documented Rheumatic Disease (*n* = 106)	No Documented Rheumatic Disease (*n* = 2844)	*p* Value
In-hospital mortality	4 (3.8%)	121 (4.3%)	0.809
30-day mortality	5 (4.7%)	158 (5.6%)	0.711
1-year mortality	10 (9.4%)	256 (9.0%)	0.879
Crude overall long-term mortality	29 (27.4%)	550 (19.3%)	0.039

Post-discharge Kaplan–Meier comparison: log-rank *p* = 0.013.

## Data Availability

The data that support the findings of this study are available from the corresponding authors upon reasonable request, subject to ethics approval and data protection restrictions.
